# Reply to: comment on “Early cancer detection by serum biomolecular fingerprinting spectroscopy with machine learning”

**DOI:** 10.1038/s41377-024-01664-2

**Published:** 2025-01-20

**Authors:** Shilian Dong, Dong He, Qian Zhang, Chaoning Huang, Zhiheng Hu, Chenyang Zhang, Lei Nie, Kun Wang, Wei Luo, Jing Yu, Bin Tian, Wei Wu, Xu Chen, Fubing Wang, Jing Hu, Xiangheng Xiao

**Affiliations:** 1 School of Physics and Technology, National Demonstration Center for Experimental Physics Education, Wuhan University, Wuhan, 430072 China; 2 Department of Laboratory Medicine, Zhongnan Hospital of Wuhan University, Wuhan, 430071 China; 3 School of Computer Science, Wuhan University, Wuhan, 430072 China; 4 Department of Hepatobiliary Pancreatic Surgery, Hubei Cancer Hospital, Tongji Medical College, Huazhong University of Science and Technology, Wuhan, 430079 China; 5 Department of Clinical Laboratory, Tianjin Medical University General Hospital, Tianjin, 300052 China; 6 Department of Blood Transfusion, Wuhan Hospital of Traditional Chinese and Western Medicine, Tongji Medical College, Huazhong University of Science and Technology, Wuhan, 430022 China; 7 Laboratory of Printable Functional Materials and Printed Electronics, Research Center for Graphic Communication, Printing and Packaging, Wuhan University, Wuhan, 430072 China; 8 Sichuan Provincial Key Laboratory for Human Disease Gene Study and the Center for Medical Genetics, Department of Laboratory Medicine, Sichuan Academy of Medical Sciences & Sichuan Provincial People’s Hospital, University of Electronic Science and Technology, Chengdu, 611731 China; 9 School of Medicine, University of Electronic Science and Technology of China, Chengdu, 611731 China

**Keywords:** Plasma physics, Optical techniques

Dear Editor,

In the accompanying Comment, Bratchenko et al. raised two concerns about the spectral data analysis pipeline employed for the Surface-enhanced Raman scattering and Artificial Intelligence for Cancer Screening (SERS-AICS) technique in our original paper^1^: (1) inappropriate accuracy presentation and (2) the use of a single data split for model evaluation. As a promising technique for molecular fingerprinting, SERS-based early cancer detection approaches using biofluids and liquid biopsy are typically evaluated based strictly on their accuracy and reliability^2–8^.

Regarding their first concern, the commenters stated that the accuracy was presented for the entire dataset instead of separating the training and test sets, leading to potential misinterpretation of the model’s performance. They provided hypothetical examples to illustrate how combining training and test data can lead to an overestimation of the model’s accuracy for early cancer prediction. In our study, we did separate training and test groups from cancer patients and healthy controls initially, using the training set to establish the AI model, which then generated the predictive values such as accuracy, sensitivity, and specificity. Adhering to the fundamental principles of building an algorithmic classification model, the training set was not subsequently used to evaluate performance. This approach avoids the high risk of overfitting, which could lead readers to misjudge the accuracy of the SERS-AICS method for pan-cancer universal screening in real-world clinical samples.

**Fig. 1:**
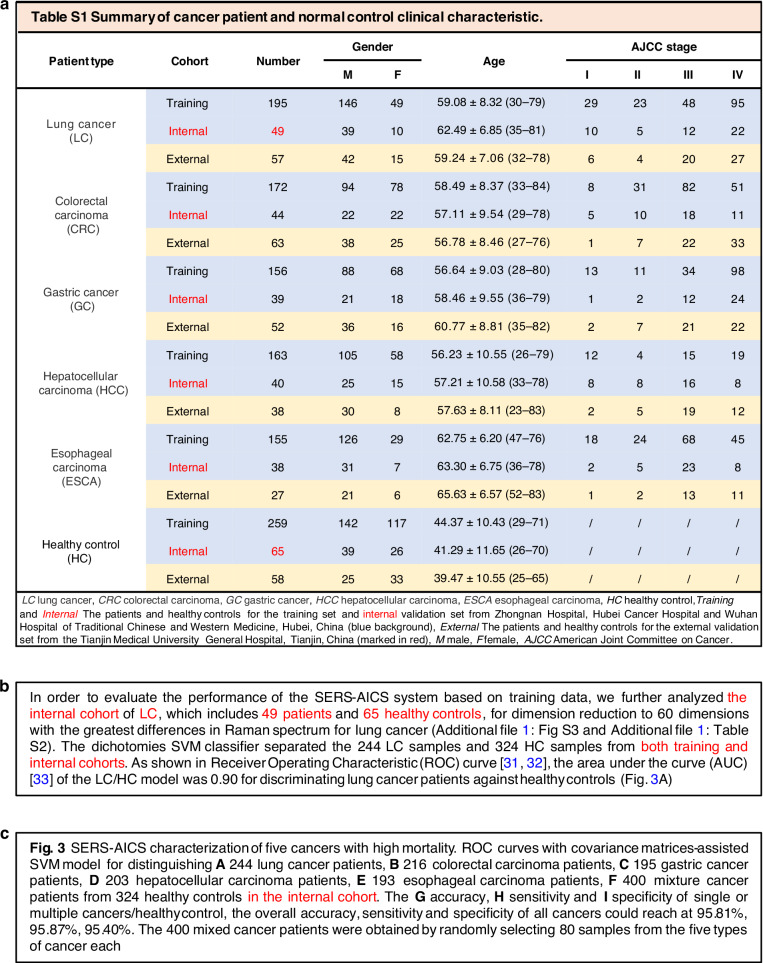
**The original usage of the term “internal” to mean “test” in various parts of the paper** **a** Supplementary Table S1. **b** Main-text (paragraph 2, lines 23–35, page 5). **c** Figure legend Fig. 3. The term “internal” and related information are highlighted in red

We firmly believe that obtaining reliable predictions requires using the test cohort exclusively, without incorporating data from the training cohort. In our original paper, we adhered to a strict workflow, as detailed in the ‘Model training’ section of the ‘Materials and Methods’ in the supporting materials, which provides more comprehensive insights than the main-text part^1^. We, however, used the term “internal” to represent “test” when referring to the real test sets in our supplementary Table S1, main-text (paragraph 2, line 25–30, page 5), and the figure legend of Fig. 3 in the original paper. This may have resulted in a misunderstanding for some readers, including the commenters (Fig. 1). In fact, our “training” set plus the “internal” set (referring to the “test” set) are equivalent to the actual internal set as typically used. We admit that the sample size and related descriptions in the original Fig. 3 may have caused some ambiguity. In the original Fig. 3a–e, the ‘*n*’ denoted the total number of samples of both the training and test cohorts. However, it is crucial to clarify here that the AUC values in Fig. 3a–e in the original paper were calculated based solely on the “internal/test” dataset. Additionally, we clearly indicated in both the figure and the corresponding figure legend, to highlight the contrast in sample sizes between the internal and external cohorts (Supplementary Fig. S9A-E in the original paper). Similarly, the calculations of accuracy, sensitivity, and specificity in Fig. 3g–i in the original paper were based exclusively on the “internal/test” cohort.

We have rechecked all our original data again and corrected some ambiguously split sample numbers in the internal or external cohorts, which resulted from the data transfer from a former team member to his successors. The small adjustments are as follows: (1) for CRC, the sample sizes of the internal training group and test group have been adjusted to 173 and 43, respectively (previously reported as 172 and 44); (2) for GC, the sample size of the external group has been updated to 53 (previously stated as 52); (3) for HCC, the sample sizes of the internal training group and test group have been updated to 162 and 41, respectively (previously stated as 163 and 40); (4) for ESCA, the sample sizes of the internal training group and test group should be 154 and 39, respectively (previously reported as 155 and 38). We will use the revised numbers for the training and test groups in the following calculations.

**Fig. 2:**
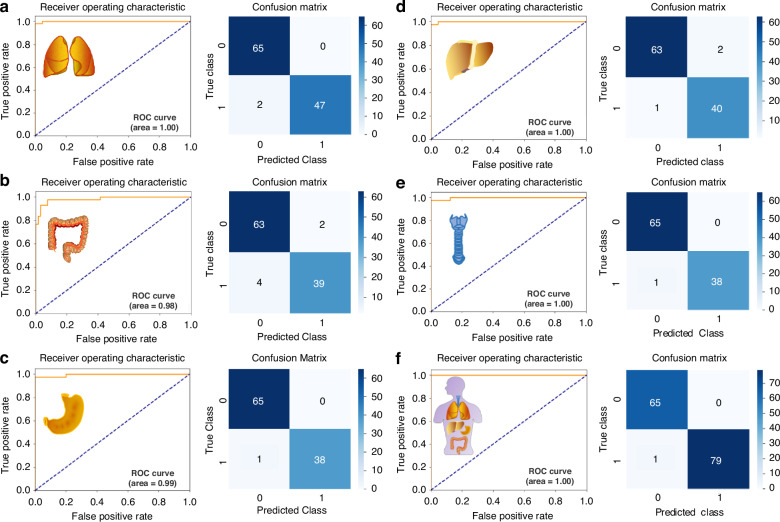
ROC curves and confusion matrix results from the test cohort of **a** LC/HC, **b** CRC/HC, **c** GC/HC, **d** HCC/HC, **e** ESCA/HC, **f** Mix/HC. Here in both axes, 0 represents the healthy control cohort, and 1 represents the disease cohort

**Fig. 3:**
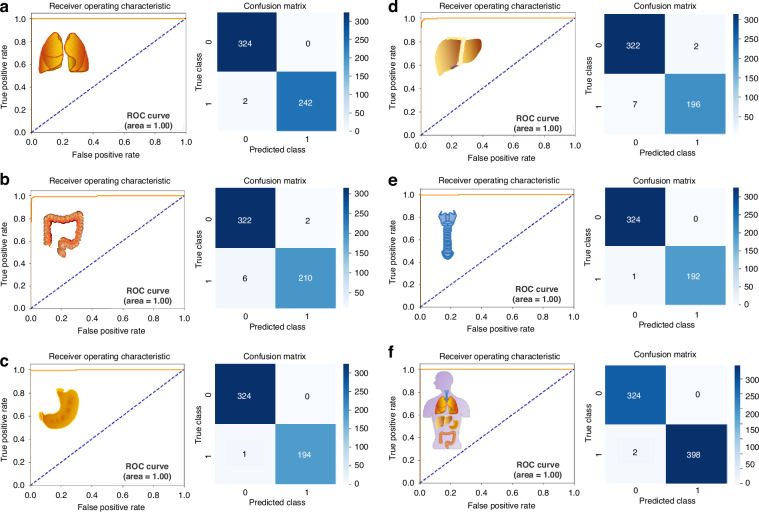
ROC curves and confusion matrix results from the training combined with test cohort of **a** LC/HC, **b** CRC/HC, **c** GC/HC, **d** HCC/HC, **e** ESCA/HC, **f** Mix/HC. Here in both axes, 0 represents the healthy control cohort, and 1 represents the disease cohort

**Fig. 4:**
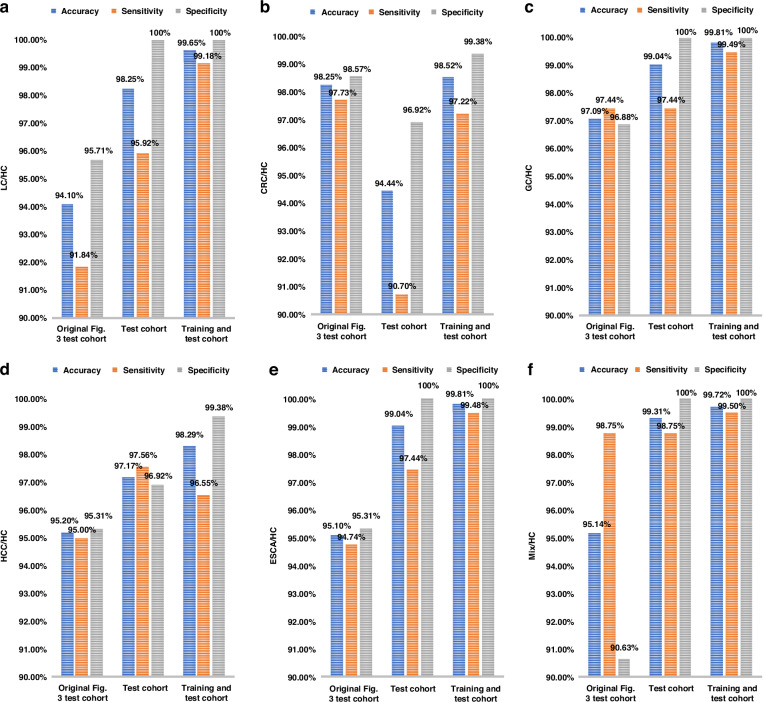
The accuracy, sensitivity, and specificity of the test cohort, the training and test cohort, and those presented in Fig. 3g–i of the original paper for **a** LC/HC, **b** CRC/HC, **c** GC/HC, **d** HCC/HC, **e** ESCA/HC, **f** Mix/HC

To provide a clearer breakdown of our data processing, we randomly re-selected training and test sets in an 8:2 ratio from our total internal cohorts. We then recalculated the ROC curves and confusion matrices for the test cohort alone (Fig. 2) and the combined training and test cohort (Fig. 3) for all five cancer types separately. Additionally, to facilitate better comparison, we generated bar charts displaying accuracy, sensitivity, and specificity for both the test cohort and the combined training and test cohort for each cancer type (Fig. 4). For the LC/HC group, the test cohort exhibited an accuracy of 98.25% and a sensitivity of 95.92% at 100% specificity, which are lower than or equal to the combined training and test cohort values of 99.65% accuracy and 99.18% sensitivity at 100% specificity (Fig. 4a). Other cancer types also exhibited similar trends (Fig. 4b–e), suggesting that the combined cohort results may reflect overfitting as the commenters emphasized. Furthermore, when compared to these re-evaluated data, our previous data of Fig. 3g–i of the original paper showed slightly lower prediction values (Fig. 4). This comparison highlights our intent not to cherry-pick better results but to demonstrate the reliability of cancer prediction using our well-established algorithm. In summary, all the results indicate that we indeed used only the test cohort (described as “internal”) for the performance evaluation of our SERS-AICS method, excluding the training cohort to avoid biased results in the original paper. This suggests that our model for early cancer prediction is both reliable and applicable.

Secondly, Bratchenko et al. also pointed out that only one split of the dataset into training and test sets was shown in our original paper, which could mask the variability and reliability of the model’s performance. They emphasized the importance of demonstrating the resulting accuracy over multiple data splits, represented as mean ± deviation/confidence interval, for a more accurate depiction of model performance.

**Fig. 5:**
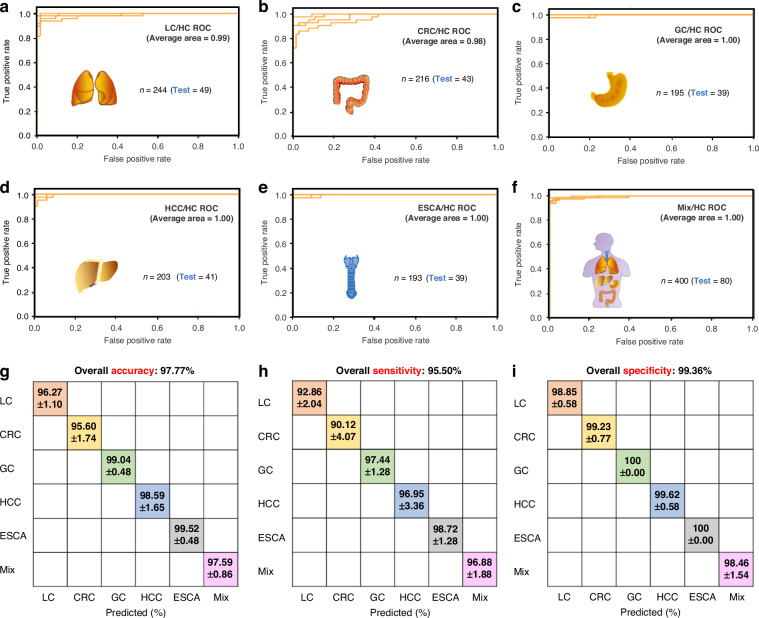
**SERS-AICS characterization of five cancers with high mortality based on internal test sets** Four representative ROC curves utilizing an SVM model with covariance matrices for the discrimination of **a** 49 lung cancer patients, **b** 43 colorectal carcinoma patients, **c** 39 gastric cancer patients, **d** 41 hepatocellular carcinoma patients, E 39 esophageal carcinoma patients, **f** a mixture of 80 cancer patients from the five types mentioned above, compared to 65 healthy controls within the internal cohort. The AUC values have been rounded to two decimal places. **g**–**i** The accuracy, sensitivity, and specificity with mean ± SD for individual or combined cancer types compared to healthy controls. The set of 400 mixed cancer patients was obtained by randomly selecting 80 samples from each of the five cancer types. Subsequently, 80 samples were randomly chosen from the total mixed pool for use in the test set, with four repetitions of random data splitting

**Fig. 6:**
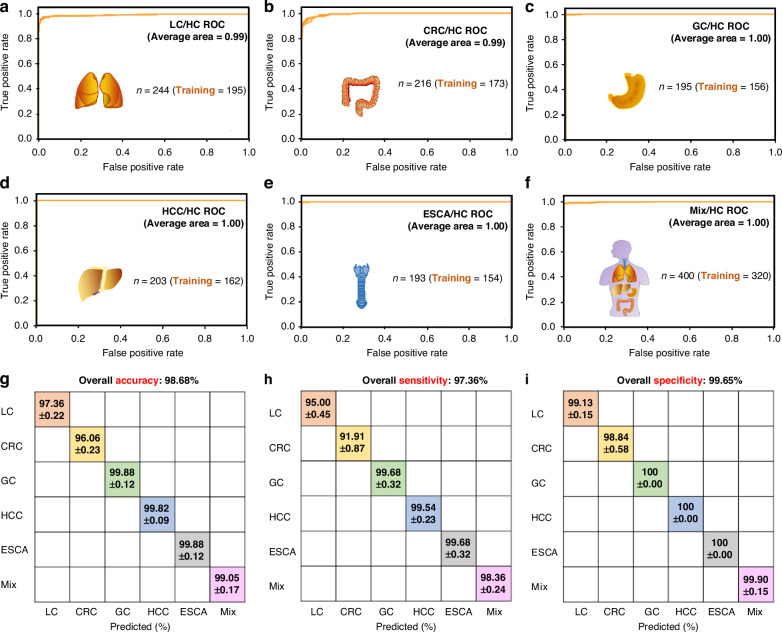
**SERS-AICS characterization of five cancers with high mortality based on internal training sets** Four representative ROC curves utilizing an SVM model with covariance matrices for the discrimination of **a** 195 lung cancer patients, **b** 173 colorectal carcinoma patients, **c** 156 gastric cancer patients, **d** 162 hepatocellular carcinoma patients, **e** 154 esophageal carcinoma patients, **f** a mixture of 320 cancer patients from the five types mentioned above, compared to 259 healthy controls within the internal cohort. The AUC values have been rounded to two decimal places. **g–i** The accuracy, sensitivity, and specificity with mean ± SD for individual or combined cancer types compared to healthy controls. The set of 400 mixed cancer patients was obtained by randomly selecting 80 samples from each of the five cancer types. Subsequently, 320 samples were randomly chosen from the total mixed pool for use in the training set, with four repetitions of random data splitting

**Fig. 7:**
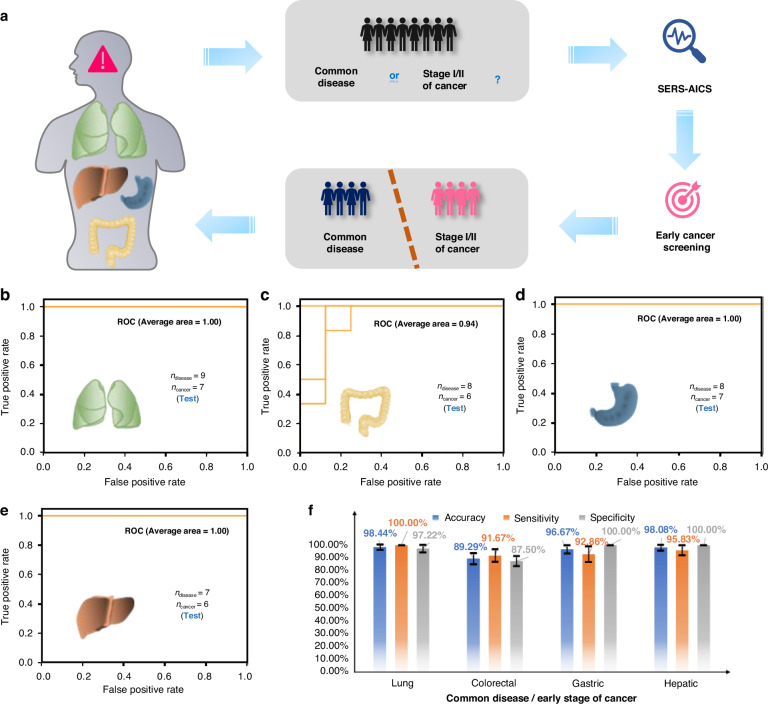
**Early screening for four representative cancers by SERS-AICS with test sets a** The SERS-AICS method demonstrates its effectiveness in distinguishing common diseases from early-stage cancers (stage I and II) with high accuracy. **b** Four ROC curves to distinguish between 9 common disease patients and 7 early-stage lung cancer patients. **c** Four ROC curves to differentiate 8 common disease patients from 6 early-stage colorectal cancer patients. **d** Four ROC curves to distinguish between 8 common disease patients and 7 early-stage gastric cancer patients. **e** Four ROC curves to discern between 7 common disease patients and 6 early-stage liver cancer patients. The AUC values have been rounded to two decimal places. **f** The accuracy, sensitivity, and specificity, along with mean ± SD values, for different common disease and early-stage cancer differentiations. The test sets were created by randomly selecting 20% of the samples from both the common disease and cancer groups

**Fig. 8:**
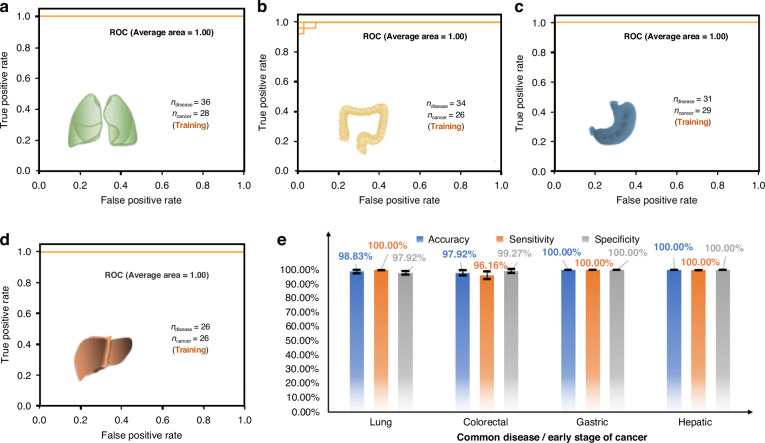
**Early screening for four representative cancers by SERS-AICS with training sets a** Four ROC curves to distinguish between 36 common disease patients and 28 early-stage lung cancer patients. **b** Four ROC curves to differentiate 34 common disease patients from 26 early-stage colorectal cancer patients. **c** Four ROC curves to distinguish between 31 common disease patients and 29 early-stage gastric cancer patients. **d** Four ROC curves to discern between 26 common disease patients and 26 early-stage liver cancer patients. The AUC values have been rounded to two decimal places. **e** The accuracy, sensitivity, and specificity, along with mean ± SD values, for different common disease and early-stage cancer differentiations. The training sets were created by randomly selecting 80% of the samples from both the common disease and cancer groups

Actually, our analysis is already unbiased, based on the random selection of a test cohort with a 20% portion from the large dataset comprising approximately 200 independent individuals for each cancer patient group. To better address the commenters’ questions regarding repetitions, we performed multiple random data splits for both the internal cohort and the early cancer/common disease cohort. This was done on an 8:2 basis, and the process was repeated a total of four times. The specific data resulting from these operations are presented in detail in Tables 1 and 2 below. In addition, the corresponding “Mean ± SD” values have been visually presented in graphs, which can be found in Figs. 5–6 for internal cohorts and Figs. 7–8 for early screening cohorts, respectively. Based on the data displayed, it is evident that the pan-cancer recognition strategy mentioned in our original paper continues to demonstrate significant recognition advantages and stability. In our manuscript, using random data segmentation only once is not the optimal data segmentation type selected to achieve the best classification results, so it can more accurately reflect the model’s ability to identify diseases. And similar practices are also reflected in some relevant literature references, including Zhang, H. et al.’s work on “Multiplexed Nanomaterial-Assisted Laser Desorption/Ionization for Pan-Cancer Diagnosis and Classification”^9^, Huang, L. et al.’s work on “Rapid, label-free histopathological diagnosis of liver cancer based on Raman spectroscopy and deep learning”^10^ and Wang. R. et al.‘s work on “Bio-Inspired In-Sensor Compression and Computing Based on Phototransistors”^11^. Therefore, we did not include standard deviation data with multiple splits of the same cohort in the original manuscript.

**Table 1:** Summary of accuracy, sensitivity, specificity, and AUC values with four repetitions from test and training sets separately within internal cohorts

**Repeat**	**Accuracy**	**Sensitivity**	**Specificity**	**AUC**	**Internal test sets of LC/HC**	**Repeat**	**Accuracy**	**Sensitivity**	**Specificity**	**AUC**	**Internal training sets of LC/HC**
1	97.37%	95.92%	98.46%	1.00		1	97.80%	95.90%	99.23%	0.99	
2	97.37%	93.88%	100.00%	0.99		2	97.36%	94.87%	99.23%	0.99	
3	95.61%	91.84%	98.46%	0.98		3	96.92%	94.36%	98.84%	0.99	
4	94.74%	89.80%	98.46%	1.00		4	97.36%	94.87%	99.23%	0.99	
Mean	96.27%	92.86%	98.85%	0.99		Mean	97.36%	95.00%	99.13%	0.99	
SD	1.10%	2.04%	0.58%	0.01		SD	0.22%	0.45%	0.15%	0.00	
Repeat	**Accuracy**	**Sensitivity**	**Specificity**	**AUC**	**Internal test sets of CRC/HC**	Repeat	**Accuracy**	**Sensitivity**	**Specificity**	**AUC**	**Internal training sets of CRC/HC**
1	95.37%	90.70%	98.46%	0.98		1	95.83%	93.06%	97.68%	0.99	
2	99.07%	97.67%	100.00%	1.00		2	95.83%	90.75%	99.23%	0.99	
3	92.59%	83.72%	98.46%	0.96		3	96.53%	92.49%	99.23%	0.99	
4	95.37%	88.37%	100.00%	0.99		4	96.06%	91.33%	99.23%	0.99	
Mean	95.60%	90.12%	99.23%	0.98		Mean	96.06%	91.91%	98.84%	0.99	
SD	1.74%	4.07%	0.77%	0.01		SD	0.23%	0.87%	0.58%	0.00	
Repeat	**Accuracy**	**Sensitivity**	**Specificity**	**AUC**	**Internal test sets of GC/HC**	Repeat	**Accuracy**	**Sensitivity**	**Specificity**	**AUC**	**Internal training sets of GC/HC**
1	99.04%	97.44%	100.00%	0.99		1	100.00%	100.00%	100.00%	1.00	
2	99.04%	97.44%	100.00%	1.00		2	99.76%	99.36%	100.00%	1.00	
3	98.08%	94.87%	100.00%	0.99		3	100.00%	100.00%	100.00%	1.00	
4	100.00%	100.00%	100.00%	1.00		4	99.76%	99.36%	100.00%	1.00	
Mean	99.04%	97.44%	100.00%	1.00		Mean	99.88%	99.68%	100.00%	1.00	
SD	0.48%	1.28%	0.00%	0.01		SD	0.12%	0.32%	0.00%	0.00	
Repeat	**Accuracy**	**Sensitivity**	**Specificity**	**AUC**	**Internal test sets of HCC/HC**	Repeat	**Accuracy**	**Sensitivity**	**Specificity**	**AUC**	**Internal training sets of HCC/HC**
1	99.06%	97.56%	100.00%	1.00		1	100.00%	100.00%	100.00%	1.00	
2	100.00%	100.00%	100.00%	1.00		2	99.76%	99.38%	100.00%	1.00	
3	95.28%	90.24%	98.46%	1.00		3	99.76%	99.38%	100.00%	1.00	
4	100.00%	100.00%	100.00%	1.00		4	99.76%	99.38%	100.00%	1.00	
Mean	98.59%	96.95%	99.62%	1.00		Mean	99.82%	99.54%	100.00%	1.00	
SD	1.65%	3.36%	0.58%	0.00		SD	0.09%	0.23%	0.00%	0.00	
Repeat	**Accuracy**	**Sensitivity**	**Specificity**	**AUC**	**Internal test sets of ESCA/HC**	Repeat	**Accuracy**	**Sensitivity**	**Specificity**	**AUC**	**Internal training sets of ESCA/HC**
1	99.04%	97.44%	100.00%	1.00		1	100.00%	100.00%	100.00%	1.00	
2	100.00%	100.00%	100.00%	1.00		2	99.76%	99.35%	100.00%	1.00	
3	99.04%	97.44%	100.00%	1.00		3	100.00%	100.00%	100.00%	1.00	
4	100.00%	100.00%	100.00%	1.00		4	99.76%	99.35%	100.00%	1.00	
Mean	99.52%	98.72%	100.00%	1.00		Mean	99.88%	99.68%	100.00%	1.00	
SD	0.48%	1.28%	0.00%	0.00		SD	0.12%	0.32%	0.00%	0.00	
Repeat	**Accuracy**	**Sensitivity**	**Specificity**	**AUC**	**Internal test sets of Mix/HC**	Repeat	**Accuracy**	**Sensitivity**	**Specificity**	**AUC**	**Internal training sets of Mix/HC**
1	96.55%	95.00%	98.46%	0.99		1	99.31%	98.75%	100.00%	1.00	
2	99.31%	98.75%	100.00%	1.00		2	98.79%	98.12%	99.61%	1.00	
3	97.24%	98.75%	95.38%	1.00		3	98.96%	98.12%	100.00%	1.00	
4	97.24%	95.00%	100.00%	1.00		4	99.14%	98.44%	100.00%	1.00	
Mean	97.59%	96.88%	98.46%	1.00		Mean	99.05%	98.36%	99.90%	1.00	
SD	0.86%	1.88%	1.54%	0.00		SD	0.17%	0.24%	0.15%	0.00

**Table 2:** Summary of accuracy, sensitivity, specificity, and AUC values with four repetitions from test and training sets separately within early screening internal cohorts

**Repeat**	**Accuracy**	**Sensitivity**	**Specificity**	**AUC**	**Common disease /Early LC (Test)**	**Repeat**	**Accuracy**	**Sensitivity**	**Specificity**	**AUC**	**Common disease /Early LC (Training)**
1	93.75%	100.00%	88.89%	1.00		1	100.00%	100.00%	100.00%	1.00	
2	100.00%	100.00%	100.00%	1.00		2	98.44%	100.00%	97.22%	1.00	
3	100.00%	100.00%	100.00%	1.00		3	98.44%	100.00%	97.22%	1.00	
4	100.00%	100.00%	100.00%	1.00		4	98.44%	100.00%	97.22%	1.00	
Mean	98.44%	100.00%	97.22%	1.00		Mean	98.83%	100.00%	97.92%	1.00	
SD	2.34%	0.00%	4.17%	0.00		SD	0.58%	0.00%	1.04%	0.00	
**Repeat**	**Accuracy**	**Sensitivity**	**Specificity**	**AUC**	**Common disease /Early CRC (Test)**	**Repeat**	**Accuracy**	**Sensitivity**	**Specificity**	**AUC**	**Common disease /Early CRC (Training)**
1	78.57%	83.33%	75.00%	0.90		1	100.00%	100.00%	100.00%	1.00	
2	85.71%	83.33%	87.50%	0.92		2	96.67%	92.31%	100.00%	1.00	
3	100.00%	100.00%	100.00%	1.00		3	95.00%	92.31%	97.06%	1.00	
4	92.86%	100.00%	87.50%	0.94		4	100.00%	100.00%	100.00%	1.00	
Mean	89.29%	91.67%	87.50%	0.94		Mean	97.92%	96.16%	99.27%	1.00	
SD	7.15%	8.34%	6.25%	0.03		SD	2.08%	3.85%	1.10%	0.00	
**Repeat**	**Accuracy**	**Sensitivity**	**Specificity**	**AUC**	**Common disease /Early GC (Test)**	**Repeat**	**Accuracy**	**Sensitivity**	**Specificity**	**AUC**	**Common disease /Early GC (Training)**
1	86.67%	71.43%	100.00%	1.00		1	100.00%	100.00%	100.00%	1.00	
2	100.00%	100.00%	100.00%	1.00		2	100.00%	100.00%	100.00%	1.00	
3	100.00%	100.00%	100.00%	1.00		3	100.00%	100.00%	100.00%	1.00	
4	100.00%	100.00%	100.00%	1.00		4	100.00%	100.00%	100.00%	1.00	
Mean	96.67%	92.86%	100.00%	1.00		Mean	100.00%	100.00%	100.00%	1.00	
SD	5.00%	10.71%	0.00%	0.00		SD	0.00%	0.00%	0.00%	0.00	
**Repeat**	**Accuracy**	**Sensitivity**	**Specificity**	**AUC**	**Common disease /Early HCC (Test)**	**Repeat**	**Accuracy**	**Sensitivity**	**Specificity**	**AUC**	**Common disease /Early HCC (Training)**
1	100.00%	100.00%	100.00%	1.00		1	100.00%	100.00%	100.00%	1.00	
2	92.31%	83.33%	100.00%	1.00		2	100.00%	100.00%	100.00%	1.00	
3	100.00%	100.00%	100.00%	1.00		3	100.00%	100.00%	100.00%	1.00	
4	100.00%	100.00%	100.00%	1.00		4	100.00%	100.00%	100.00%	1.00	
Mean	98.08%	95.83%	100.00%	1.00		Mean	100.00%	100.00%	100.00%	1.00	
SD	2.88%	6.25%	0.00%	0.00		SD	0.00%	0.00%	0.00%	0.00

**Fig. 9:**
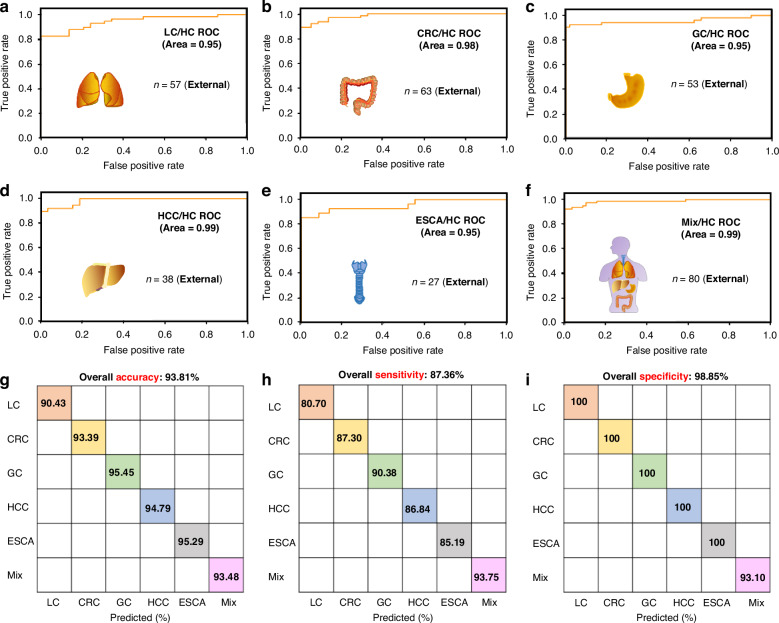
**SERS-AICS characterization of five cancers with high mortality based on external sets** ROC curves with covariance matrices-assisted SVM model for distinguishing **a** 57 lung cancer patients, **b** 63 colorectal carcinoma patients, **c** 53 gastric cancer patients, **d** 38 hepatocellular carcinoma patients, **e** 27 esophageal carcinoma patients, **f** 80 mixture cancer patients from 58 healthy controls in the internal cohort. The **g** accuracy, **h** sensitivity, and **i** specificity of single or multiple cancers/healthy control, the overall accuracy, sensitivity, and specificity of all cancers could reach 93.81%, 87.36%, and 98.85%

In addition to the internal cohorts, we have also re-executed the algorithm while implementing optimized processing techniques for the external cohorts. Upon comparing the original data with the revised data, it is evident that, with the exception of the sensitivity values for LC, GC, HCC, and ESCA, which experienced slight reductions in the revised external dataset, the majority of values demonstrated significant improvements in cancer prediction when utilizing our AICS algorithm. This comparative analysis is presented in Table 3 and Fig. 9.

**Table 3:** A Comparative summary of accuracy, sensitivity, and specificity values within external cohorts between the original data and the revised data

Accuracy	Original data (external)	Revised date (external)
LC	84.35%	90.43%
CRC	80.33%	93.39%
GC	83.64%	95.45%
HCC	84.38%	94.79%
ESCA	87.06%	95.29%
**Sensitivity**	**Original data (external)**	**Revised date (external)**
LC	93.10%	80.70%
CRC	86.21%	87.30%
GC	93.10%	90.38%
HCC	93.10%	86.84%
ESCA	96.55%	85.19%
**Specificity**	**Original data (external)**	**Revised date (external)**
LC	75.44%	100.00%
CRC	75.00%	100.00%
GC	73.05%	100.00%
HCC	71.05%	100.00%
ESCA	66.67%	100.00%

In summary, we addressed the commenters’ concerns by (1) clarifying the accuracy of our cancer screening AI algorithm was evaluated solely based on the test cohort, not combined with the training cohort, to avoid overfitting; and (2) demonstrating the reliability for our cancer prediction model by presenting consistent results even across multiple data splits. All the intermediate and final data presented here provide detailed and transparent explanations of the methodologies and results, ensuring the accuracy and reliability of our SERS-AICS for future applications.
